# Exploring the Effects of Process Parameters during W/O/W Emulsion Preparation and Supercritical Fluid Extraction on the Protein Encapsulation and Release Properties of PLGA Microspheres

**DOI:** 10.3390/pharmaceutics16030302

**Published:** 2024-02-21

**Authors:** Heejun Park

**Affiliations:** College of Pharmacy, Duksung Women’s University, 33, Samyangro 144-gil, Dobong-gu, Seoul 01369, Republic of Korea; heejunpark@duksung.ac.kr; Tel.: +82-2-901-8589

**Keywords:** supercritical fluid extraction of emulsions, PLGA microspheres, encapsulation efficiency, initial burst release, bovine serum albumin

## Abstract

In this study, protein-loaded poly(lactic-co-glycolic acid) (PLGA) microspheres were prepared via supercritical fluid extraction of emulsion (SFEE) technology. To understand the correlation between process parameters and the main quality characteristics of PLGA microspheres, a comprehensive prior study on the influence of process variables on encapsulation efficiency (EE), initial drug burst release (IBR), morphology, surface property, and particle size distribution (PSD) was conducted within a wide process condition range of each unit process step, from the double-emulsion preparation step to the extraction step. Bovine serum albumin (BSA), a high-molecular weight-protein that is difficult to control the IBR and EE of PLGA microspheres with, was used as a model material. As double-emulsion manufacturing process parameters, the primary (W/O) and secondary emulsion (W/O/W) homogenization speed and secondary emulsification time were evaluated. In addition, the effect of the SFEE process parameters, including the pressure (70–160 bar), temperature (35–65 °C), stirring rate (50–1000 rpm), and flow rate of supercritical carbon dioxide, SC-CO_2_ (1–40 mL/min), on PLGA microsphere quality properties were also evaluated. An increase in the homogenization speed of the primary emulsion resulted in an increase in EE and a decrease in IBR. In contrast, increasing the secondary emulsification speed resulted in a decrease in EE and an increase in IBR along with a decrease in microsphere size. The insufficient secondary emulsification time resulted in excessive increases in particle size, and excessive durations resulted in decreased EE and increased IBR. Increasing the temperature and pressure of SFEE resulted in an overall increase in particle size, a decrease in EE, and an increase in IBR. It was observed that, at low stirring rates or SC-CO_2_ flow rates, there was an increase in particle size and SPAN value, while the EE decreased. Overall, when the EE of the prepared microspheres is low, a higher proportion of drugs is distributed on the external surface of the microspheres, resulting in a larger IBR. In conclusion, this study contributes to the scientific understanding of the influence of SFEE process variables on PLGA microspheres.

## 1. Introduction

Controlling drug encapsulation efficiency (EE), particle size distribution (PSD), and drug release profile is important in using PLGA microspheres for a sustained-release (SR) drug delivery system [1,2,3,4]. Especially, excessive initial drug burst release (IBR) can increase the risk of adverse effects due to overdose [5,6,7,8,9,10,11].

Among the emulsion-based PLGA microsphere-manufacturing technologies, including solvent extraction, solvent evaporation (SE), coacervation, and spray drying to achieve this drug release control purpose [3,12,13,14,15], recent research on poly (lactic/glycolic) acid (PLGA) microsphere manufacturing using a new technology called supercritical fluid extraction of emulsions (SFEEs) has been pioneered by several leading teams [16,17,18,19,20,21,22,23,24,25,26,27,28,29]. Typically, the SFEE process for the preparation of PLGA microspheres involves two major steps: the preparation of the emulsion and the fabrication/solidification of PLGA via organic solvent removal [16]. It has been widely reported that the influence of double (W/O/W)-emulsion manufacturing process variables on the quality characteristics of the final fabricated PLGA microspheres is important. However, many studies on using the SFEE process to achieve PLGA microspheres for SR have been conducted focusing only on the extraction process of SFEE, without understanding the influence of emulsification process variables on the outcomes. In other words, studies proceed directly to the SFEE stage without optimizing the emulsification process. Therefore, it is necessary to conduct research on moving to the SFEE stage with emulsions manufactured under optimized emulsification process conditions after conducting preliminary research on the screening and optimization of the influence of process variables in the double-emulsion preparation stage before the extraction stage. 

Considering the abovementioned limitations in the field of SFEE research, this study was aimed at understanding the correlation between the process parameters and main quality characteristics of PLGA microspheres. To achieve this goal, a comprehensive prior study on the influence of process variables on EE and IBR, the most important quality characteristics of PLGA microspheres, was conducted within a wide process condition range of each unit process step, from the double-emulsion preparation process to the SFEE process. BSA, a high-molecular-weight protein that is difficult to control the IBR and EE of PLGA microspheres with, was used as a model material due to the advantages of its widespread availability, low cost, and high purity. In addition, we also aimed to scientifically examine the impact of process variables through a combination of not only EE and IBR but also morphology, surface properties, and PSD. As double-emulsion manufacturing process parameters, the primary (W/O) and secondary emulsion (W/O/W) homogenization speed and secondary emulsification time were evaluated. Through this, the double emulsion manufactured under selected conditions was applied to SFEE to evaluate the effect of the SFEE process parameters, including the pressure, temperature, stirring rate, and flow rate of SC-CO_2_, on PLGA microsphere quality properties. These main process parameters for emulsification and extraction stages were chosen based on many scientific references applying SFEE in various fields [18,30,31,32,33,34,35,36,37].

## 2. Materials and Methods

### 2.1. Materials

PLGA 50/50 was obtained from Boehringer-Ingelheim (Resomer 502H, Ingelheim am Rhein, Germany). BSA, sucrose and poly(vinyl alcohol) (PVA, M.W. 13,000–23,000), tween 20, and sodium azide were purchased from Sigma Aldrich (Saint Louis, MO, USA). Phosphate-buffered saline (PBS) was obtained from Welgene (Gyeongsan, Republic of Korea). CO_2_ with a high purity of 99.99% was obtained from Hanmi Gas Co. Ltd. (Anseong, Republic of Korea). Dichloromethane (DCM), acetonitrile, tetrahydrofuran (THF), and acetone were purchased from Merck (Fair Lawn, NJ, USA). All other chemicals were of reagent grade and used without further purification. 

### 2.2. Changes in Process Parameters at Double (W/O/W)-Emulsion Preparation Stage

The effect of changes in W/O/W emulsion manufacturing process variables on the characteristics of BSA-loaded PLGA microspheres was conducted within the range of process conditions shown in Table 1 below. Considering experimental efficiency, the prepared double emulsion was solidified through the solvent evaporation (SE) method, and characteristics of microspheres were evaluated to determine the correlation with double-emulsion manufacturing process parameters. To prepare the inner water phase, 5 mg of BSA and 20 mg of sucrose were dissolved in deionized water (D.W., 100 μL). The oil phase was prepared by dissolving 200 mg of PLGA 50:50 (RG 502H) in 2.5 mL of DCM. The inner aqueous phase was added to the oil phase, then homogenized (ULTRA-TURRAX^®^ T-18 Basic, IKA^®^-WERKE GMBH, Stauffen, Germany) at various speeds (from 6500 to 24,000 rpm) for 5 min. This primary emulsion was introduced into 12.5 mL of 1% PVA aqueous solution, then emulsified to prepared the secondary double emulsion (W/O/W), at various rpm (from 6500 to 17,500 rpm) and various time periods (from 0.5 to 4 min). Additionally, 12.5 mL of 1% PVA solution was added in the double emulsion. The PLGA was solidified via the removal of the volatile organic solvent in the W/O/W emulsion using an N-1110 rotary evaporator under vacuum (EYELA, Tokyo, Japan) at 35 °C and 150 rpm. The obtained PLGA microsphere suspensions were washed three times with a sterilized solution for injection (0.9% NaCl and 5% dextrose) via 600× *g* centrifugation (Eppendorf centrifuge 5019R, Hamburg, Germany) followed by supernatant removal. The collected PLGA microspheres were frozen in a deep freezer, then dried through lyophilization using an FD8508 freeze-dryer (Ilshin BioBase Co. Ltd., Dongducheon, Republic of Korea).

### 2.3. Changes in Process Parameters at SFEE Process Stage 

**Figure 1:**
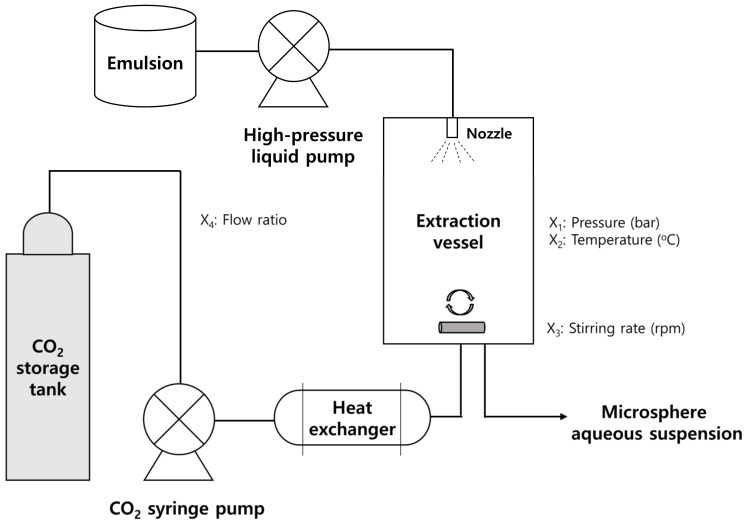
A diagram illustrating the apparatus for the supercritical fluid extraction of emulsions (SFEEs) (adopted with permission from [39]).

PLGA microspheres loaded with BSA were prepared utilizing SFEE technology under various process conditions to evaluate how alterations in process variables affect the properties of PLGA microspheres. The process conditions outlined as 5-1 in Table 1, designated for the preparation of the double emulsion, were identified as the most suitable ones and subsequently utilized to prepare the double emulsions for applications in SFEE. A diagram outlining the SFEE apparatus employed in the preparation of microspheres is presented in Figure 1 [38].

In the initial step, liquid CO_2_ was transferred from the storage tank to the preheater. The liquid CO_2_ was then heated and compressed before being transported to the cylindrical stainless-steel extraction vessel, which had a volume of approximately 70 mL. The conveyance of CO_2_ was accomplished via the a spray nozzle until the intended pressure level was reached utilizing an ISCO syringe pump (Model 260D, Lincoln, NE, USA). Upon achieving an equilibrium of pressure and temperature under the specified conditions, the prepared W/O/W double emulsion (15 mL) was introduced into the high-pressure extraction chamber via a liquid pump (Model 307, Gilson Inc., Middleton, WI, USA). Subsequently, SC-CO_2_ was continuously supplied to the vessel at a consistent flow rate to extract the used volatile organic solvent from the W/O/W emulsion utilizing a backpressure regulator (Tescom, model 26-1723-24-194, Minneapolis, MN, USA) under constant process conditions of pressure and temperature. During the extraction process, the emulsion was agitated utilizing a magnetic stirrer to enhance diffusion and prevent the coalescence of the emulsion. Throughout the SFEE process, the primary process parameters including pressure, temperature, stirring rate, and SC-CO_2_ flow rate were systematically adjusted within the ranges of 35–65 °C, 70–160 bar, 50–1000 rpm, and 1–40 mL/min, respectively. These selected factors are widely recognized as critical process parameters in SFE processes across diverse application fields [18,30,31,32,33,34,35,36,37]. After completing the extraction process, the chamber was gradually depressurized to reach atmospheric pressure. The prepared PLGA microspheres in the suspensions were collected, then washed and freeze-dried as described above.

### 2.4. Characterization of BSA-Loaded PLGA Microsphere

#### 2.4.1. Morphology Analysis

The size and morphologies of the emulsion and suspended microspheres were evaluated via optical microscopy (ZEISS Obser. D1, Carl Zeiss Microimaging GmbH, Oberkochen, Germany). In addition, scanning electron microscopy (SEM, JSM-6700F, JEOL, Tokyo, Japan) was also used to characterize the morphology of the prepared microsphere. The dried samples of microsphere powder were dispersed onto a metal support stub affixed with double-sided conductive carbon adhesive tape. Subsequently, the microspheres underwent gold palladium coating via an automated sputter coater. SEM analysis was conducted at an accelerating voltage of 5 kV, capturing multiple images at suitable magnifications.

#### 2.4.2. Analysis of Particle Size Distribution

The particle size and particle distribution in the primary (W/O) emulsion were determined via the dynamic light scattering (DLS) method (ELS-Z, Otsuka Electronics, Osaka, Japan), which is suitable for particle size analysis in the size range of nanoparticles to those of a few microns in size (available particle size measurement range according to the specifications of equipment: 0.1~10,000 nm). The particle size and particle distribution of the secondary (W/O/W) emulsion and freeze-dried microspheres were determined via laser diffractometry using Mastersizer^TM^ (MS2000, Malvern, UK), which is more suitable for particle size analysis in the micro-size range (available particle size measurement range according to the specifications of equipment: 0.02–2000 µm). The particle size was expressed as the volume mean diameter (VMD). The size distribution was assessed using the SPAN value, calculated as the ratio of D_90%_–D_10%_ to D_50%_, where D_N%_ (N = 10, 50, 90) denotes the volume percentage of microspheres with diameters up to D_N%_ equivalent to N%. A smaller SPAN value indicates a narrower particle size distribution.

#### 2.4.3. Dynamic Vapor Sorption (DVS)

DVS analysis was performed using a humidity-controlled microbalance (DVS, Surface Measurement Systems, Wembley, UK) to investigate the moisture sorption behavior of the prepared PLGA microsphere. Approximately 15 mg of the samples was placed on the sample holder and then equilibrated to a relative humidity (RH) of <1%. The RH was incrementally raised from 0% to 90% RH in increments of 10% RH for adsorption isotherm analysis. With every incremental change in RH, the system regulated the RH and monitored the sample weight until equilibrium conditions were attained. Subsequently, the equilibrium weight and temperature at each relative humidity step were recorded. If the change in weighted mass was below 0.01% within a 40 min interval, the subsequent step was executed. 

#### 2.4.4. Analysis of Residual Organic Solvent in PLGA Microspheres

The residual concentrations of organic solvent (DCM) on PLGA microspheres were determined using gas chromatography (GC). The lyophilized PLGA microspheres were accurately weighed and completely dissolved in 1 mL of THF. Following this, 4 mL of methanol was introduced into the solution, and the mixture was agitated using a vortexer for 1 min to induce the precipitation of the PLGA polymer. Then, precipitated PLGA microspheres were separated using a centrifuge at 12,000 rpm for 10 min (1730 MR, GYROZEN Co. Ltd., Daejeon, Republic of Korea). To analyze the residual solvent, the supernatant collected after centrifugation was injected into the GC system (Hewlett and Packard, 5890 SERIES 2, Palo Alto, CA, USA) coupled with a flame ionization detector (GC-FID). Acetone was employed as the internal standard. The residual solvent was separated using a fused-silica capillary column (Supelco, SPB™-1, Bellefonte, PA, USA) with a length of 30 m, an internal diameter of 0.53 mm, and a film thickness of 0.5 μm. The analysis conditions of GC included an oven temperature of 40 °C maintained for 8 min. The injector temperature was maintained at 180 °C, and helium carrier gas (approximately 2 mL/min) was employed at a pressure and a split flow rate of 65 kPa and 6 mL/min, respectively.

#### 2.4.5. Encapsulation Efficiency (EE)

To extract BSA from the prepared PLGA microspheres, microsphere samples containing 10 mg of BSA were dissolved in 3 mL of acetonitrile via agitation using a vortexer. Following this, 3 mL of acetate buffer solution (pH 4.5, 30 mM) was swiftly added and agitated for PLGA polymer precipitation. The resulting suspension, subsequent to the removal of acetonitrile via rotary evaporation under vacuum, underwent centrifugation at 12,000 rpm for 5 min using a microcentrifuge (model 1730 MR, GYROZEN Co. Ltd., Gimpo, Republic of Korea) to isolate the precipitated PLGA polymer. The collected supernatant was injected into the HPLC equipped with a size exclusion chromatography (SEC) column (TSK-GEL^®^, 7.8 mm × 30 cm, TOSOH BIOSEP PART #08540) for BSA quantification. This SEC analyses were conducted using an Agilent 1290 Infinity HPLC system (Waldbronn, Germany), which comprises a pump (Model 1260 Quat Pump VL), an autosampler (Model 1260 ALS), and a UV detector (Model 1260 VWD DL). The auto-sampler and column temperature were maintained at 5 °C and 30 °C, respectively, throughout the analyses. The mobile phase was prepared with a composition of 0.15 M sodium chloride and 50 mM sodium phosphate (pH 7.25). The UV detection wavelength and flow rate of the mobile phase were set to 214 nm and 0.8 mL/min, respectively. The EE values were obtained using Equation (1):

EE (%) = (Measured LC/Theoretical LC) × 100(1)

where LC represents the loading capacity percentage, calculated by multiplying the mass of BSA contained within the microspheres by 100, divided by the mass of the microspheres.

#### 2.4.6. In Vitro BSA Release Test 

Tween 20 (0.02% *w*/*v*) and sodium azide (0.01% *w*/*v*) were dissolved in pH 7.4 phosphate-buffered saline (PBS), serving as a dispersing and preserving agent, respectively. This medium was used for the drug release test. Precisely weighed PLGA microspheres were added to 2 mL of drug release medium within a glass tube. After the commencement of incubation in a shaking water bath set at 37 °C and 100 rpm, the drug-released medium was retrieved from samples after centrifugation (4000 rpm for 5 min) at predetermined intervals, and an equivalent volume of fresh medium was then added. The collected medium containing released BSA underwent centrifugation at 12,000 rpm and 5 °C for 10 min to separate the undissolved fine material and yield a clear supernatant suitable for HPLC analysis, following the HPLC method described above. The initial burst release (IBR) was determined based on the percentage of BSA measured on the first day.

### 2.5. Statistical Analysis

Statistical significance was assessed using either an independent *t*-test or a one-way analysis of variance (ANOVA) test. All statistical analyses were performed using SPSS v12.0 software (IBM SPSS, Chicago, IL, USA).

## 3. Results and Discussion

### 3.1. Effect of Process Parameters during W/O/W Emulsion Preparation

#### 3.1.1. Effect of Primary (W/O) Emulsion Homogenization Speed

**Figure 2:**
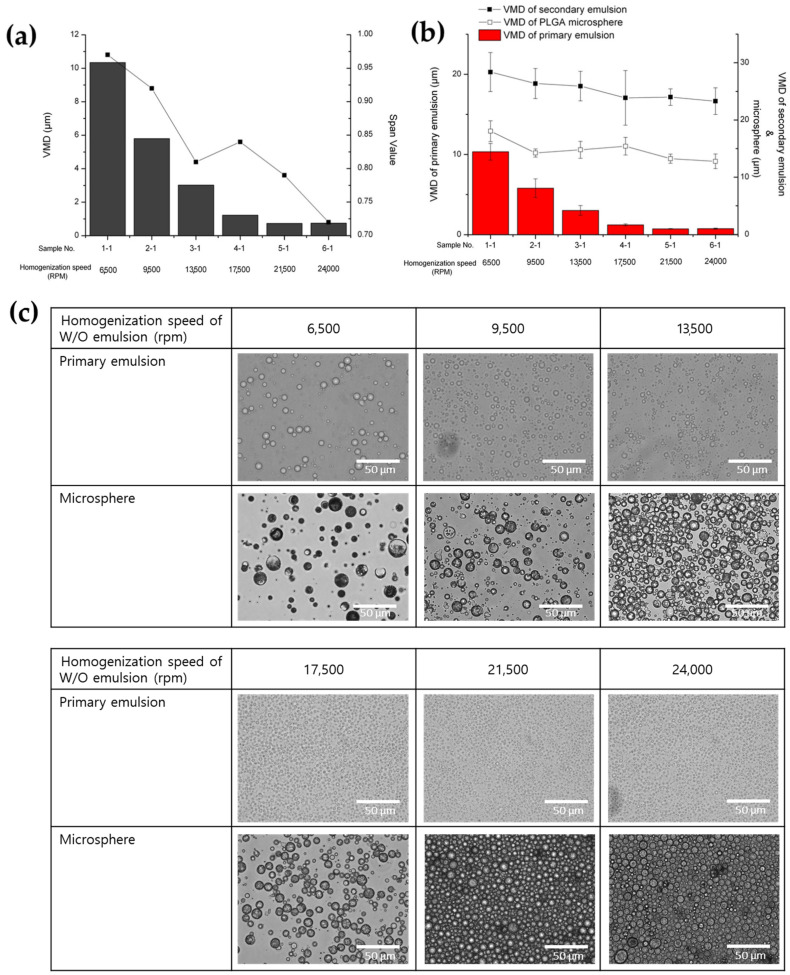
The influence of the primary (W/O) emulsion homogenization speed on (**a**) the particle size and SPAN value of the primary emulsion, (**b**) the particle size of the secondary emulsion and fabricated PLGA microspheres, and (**c**) optical microscopic images of the primary emulsions and PLGA microspheres.

**Figure 3:**
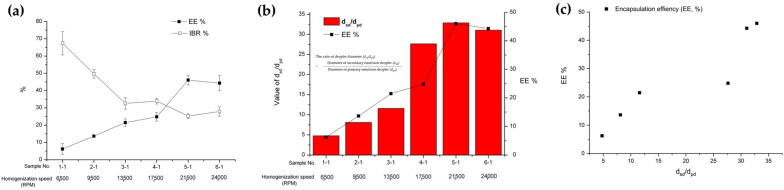
The effect of primary (W/O) emulsion homogenization speed on (**a**) the EE and IBR of the BSA-loaded PLGA microsphere, (**b**) the ratio of the droplet diameter (d_sd_/d_pd_) of the double emulsion, and (**c**) the relationship between d_sd_/d_pd_ and EE.

The influence of primary emulsion homogenization speed on the particle size of the primary emulsion, secondary emulsion, and PLGA microspheres was examined. Additionally, the EE and IBR of the prepared PLGA microspheres were evaluated (Table 1, Figure 2 and Figure 3). The homogenization speed during the preparation of the primary emulsion significantly impacted both the diameter and SPAN value of the primary emulsion. Specifically, higher homogenization speeds were associated with smaller particle sizes and a more uniform distribution (Figure 2a). However, its impact on the droplet diameter of the secondary emulsion and the particle size of the microspheres was found to be negligible (Figure 2b). It has been reported that, generally, the stirring rate employed in the preparation of the primary emulsion does not have a significant impact on the size of the microspheres [40]. In contrast, it showed a tendency for there to be a proportional relationship between the size of the secondary emulsion and the size of the microspheres. The particle size of the fabricated PLGA microspheres is directly correlated with the final emulsion droplet size and is thus contingent upon the formulation, dispersion method and condition, and the stability of the emulsion [41,42]. The results were confirmed through optical microscopic observation (Figure 2c).

Interestingly, a significant impact of primary emulsion homogenization speed on both the EE and IBR was observed. (Figure 3a,b). The preparation at a higher homogenization speed during the primary emulsion preparation stage resulted in higher EE. It has been reported that microspheres exhibiting high EE are obtained from smaller primary emulsions [40,43]. In addition, as shown in Figure 3b,c, the ratio of the droplet diameter (d_sd_/d_pd_) of the secondary emulsion (d_sd_) to that of the primary emulsion (d_pd_) seemed to strongly affect the EE of BSA in the prepared PLGA microspheres [44]. Overall, it was shown that achieving a higher EE is more probable with an increase in d_sd_/d_pd_. From this result, it is suggested that the small value of d_sd_/d_pd_ could mean that a thin oil layer existed around the primary emulsion, and that this thin oil layer facilitated the leakage of the inner phase into the outer water phase.

#### 3.1.2. Effect of Secondary (W/O/W) Emulsion Homogenization Speed

**Figure 4:**
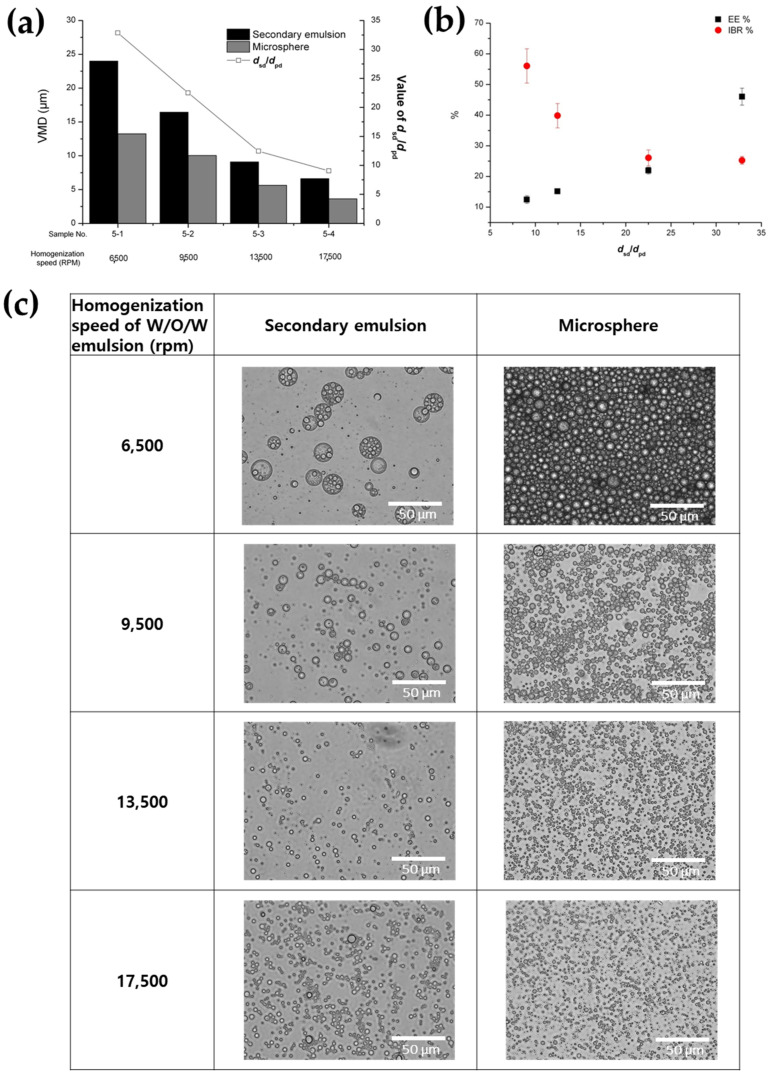
The effect of secondary (W/O/W) emulsion homogenization speed on (**a**) the particle size of the secondary double emulsion and microsphere, (**b**) the ratio of the droplet diameter (d_sd_/d_pd_) of the double emulsion, and (**c**) optical microscopic images of the secondary double emulsions and PLGA microspheres.

The influence of homogenization speed during the preparation of the secondary emulsion was studied (Table 1 and Figure 4). There was a significant effect of homogenization speed during the preparation of the secondary emulsion on its diameter. The diameters of the secondary emulsion exhibited a strong correlation with the particle sizes of the microspheres, indicating a tendency toward a proportional relationship between them (Figure 4a). As shown in Figure 4a, the value of d_sd_/d_pd_ decreased with an increase in homogenization speed during the preparation of the secondary emulsion [43,44]. In addition, a significant impact of the secondary emulsion homogenization speed on the EE and IBR of BSA from the PLGA microspheres was observed (Figure 4b). A lower EE was induced through preparation with a higher homogenization speed during the preparation of the secondary emulsion. It was identified again that the microspheres with high EE were obtained when the value of d_sd_/d_pd_ was large. Moreover, the IBR of BSA within the first day of drug release increased at higher homogenization speeds during the preparation of the secondary emulsion. As mentioned above, it is hypothesized that the presence of a thin oil layer around the primary emulsion, indicated by a small value of d_sd_/d_pd_, facilitated the leakage of the inner phase into the outer water phase, thereby increasing the amount of surface-exposed BSA. This overall negative phenomenon may have led to a decrease in EE and an increase in IBR [45].

#### 3.1.3. Effect of Homogenization Time Period of Secondary (W/O/W) Emulsion

**Figure 5:**
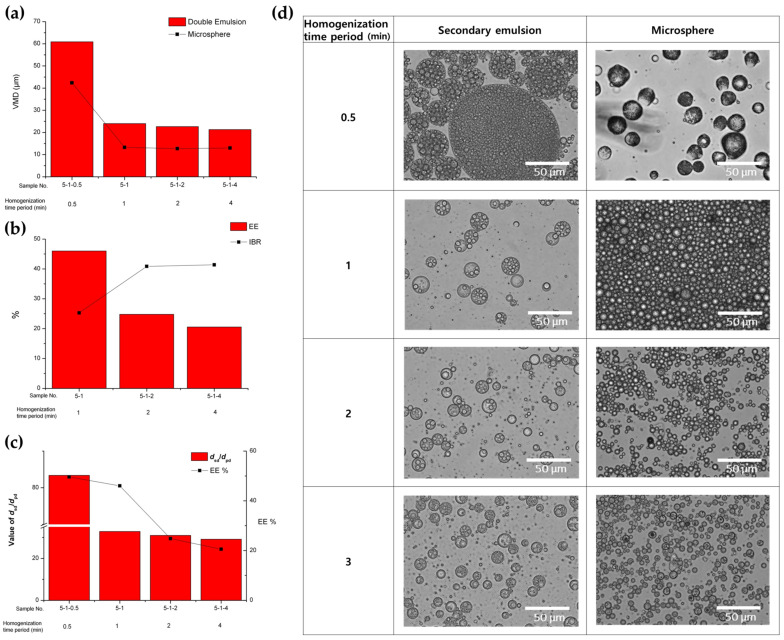
The effect of the homogenization time period during the preparation of the secondary (W/O/W) emulsion on (**a**) the size of the double emulsion and PLGA microsphere, (**b**) the EE and IBR of the PLGA microspheres, (**c**) the ratio of the droplet diameter (d_sd_/d_pd_) of the double emulsion, and (**d**) optical microscopic images of the secondary double emulsions and PLGA microspheres.

The effect of the homogenization time period during the preparation of the secondary emulsion was investigated under conditions where the time period varied between 0.5 and 4 min, with homogenization speeds set at 21,500 and 6500 rpm for the primary and secondary emulsions, respectively (Table 1 and Figure 5). The secondary emulsion prepared in a time period of 0.5 min of homogenization had a very large size (VMD = 60.88 µm) with an irregular size distribution (SPAN value = 1.86) (Figure 5a,d). This observation may be attributed to the inadequate mixing efficiency resulting from an insufficient homogenization time. On the other hand, the size of the secondary emulsions was not significantly affected by a homogenization time period between 1 and 4 min, although the span value was slightly decreased as a result of the increase in the homogenization time period (Table 1 and Figure 5a). In addition, it was confirmed again that the secondary emulsion size was well correlated proportionally with the solidified microsphere size. The homogenization time period during the secondary emulsification stage had a significant effect on both the EE and IBR of the BSA loaded onto the microspheres (Figure 5b,c). As the homogenization time period increased, the EE decreased. Additionally, the IBR of BSA increased with longer homogenization time periods. This negative result may be attributed to the increased diffusion of BSA out of the inner aqueous phase and the elevated surface exposure of BSA as the homogenization time period was prolonged. Furthermore, Figure 5c shows that, overall, microspheres with high EE were obtained when the value of d_sd_/d_pd_ was large, consistent with the results presented above. However, interestingly, when comparing sample 5-1 with other samples for which the stirring time was increased, the value of d_sd_/d_pd_ was similar, but the EE increased remarkably. This result suggests that increasing the stirring time could result in the leakage of the inner phase to outer water phase to easily occur through the DCM oil layer.

### 3.2. Effect of Process Parameters during SFEE Operating

#### 3.2.1. Effect of Pressure 

**Figure 6:**
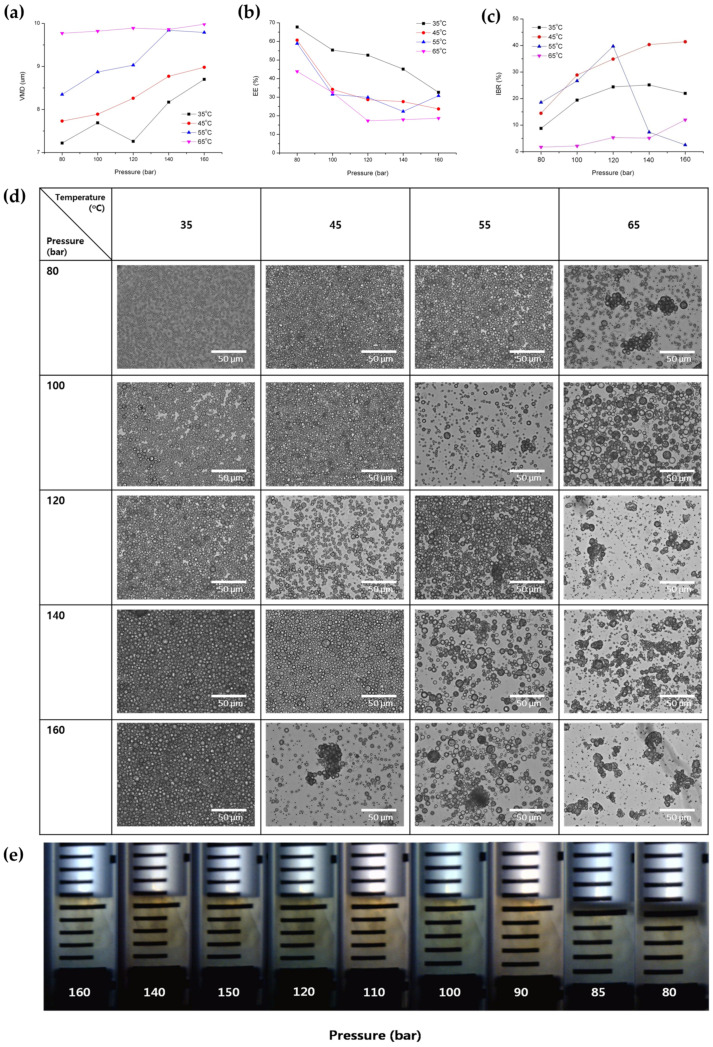
The effects of pressure and temperature during SFEE on (**a**) the particle size, (**b**) EE, (**c**) IBR, and (**d**) morphology of BSA-loaded PLGA microspheres, and (**e**) the phase transitions including the glass transition and solidification of PLGA resulting from alterations in pressure under supercritical conditions.

As depicted in Table 2 and Figure 6, it was observed that, overall, the particle size of PLGA microspheres increased with increasing pressure. This result can be explained by an earlier study, which suggested that the volume of the DCM droplet steadily increases until a dense thin layer of PLGA is fabricated during SFEE conducted at relatively high pressures [46]. 

Figure 6b shows a trend of the EE decreasing as pressure increased above 80 bar. The pressure applied during the SFEE process influences both the transfer rates of SC-CO_2_ and DCM, and the mass transfer of the drug molecule within the inner aqueous phase. In cases of excessively high pressure during the SFEE process, the enhanced diffusion rate of the encapsulated drug could result in a reduction in EE. This phenomenon arises from the increased probability of the drug migrating from the inner aqueous phase to the outer aqueous phase due to increased mass transfer [41,47,48]. The elevated pressure within the hydrated PLGA microspheres during the SFEE process can instigate pore formation and/or microsphere breakage owing to the sudden diffusion of SC-CO_2_ from the polymer layer. Consequently, this phenomenon may lead to diminished EE. This explanation was confirmed via the optical microscopic image analysis, which allowed us to observe phase transitions including the glass transition and solidification of PLGA resulting from alterations in pressure under supercritical conditions (Figure 6d). This negative impact on EE can be attributed to the elevated pressure conditions, which may have induced the glass transition of the PLGA polymer. This transition occurred as the dense outer layer of the PLGA microspheres became more flexible under increased pressure. Several reports suggested that the SC-CO_2_ has the effects of “glass transition temperature (T_g_) lowering” on PLGA, which result in increased drug loss from the PLGA microspheres during the SFEE process [1,49,50,51,52]. The optical microscopic image analysis of the PLGA glass transition in SC-CO_2_ (Figure 6e) represents a “T_g_ lowering” of PLGA under SC conditions of various high pressures at 35 °C. After placing PLGA powder into a tube with a graduated gauge, it was positioned within the vessel to allow for a clear observation of the PLGA state, as depicted in Figure 6e. Subsequently, supercritical carbon dioxide (SC-CO_2_) was injected to achieve a pressure equilibrium of 160 bar at 35 °C. Due to the T_g_-lowering effect of SC-CO_2_ mentioned above, PLGA subjected to supercritical conditions under high pressure underwent a transition from a solid to a viscous state, appearing as an opaque continuous phase without voids between particles. Then, the pressure was gradually reduced to 80 bar with equilibration for 12 h at each pressure stage. Interestingly, it was shown that the solidification of the PLGA surface was started from around 85 bar, and the area of the solidified surface gradually expanded over time when the pressure was further reduced to 80 bar. From this result, it was suggested that the solidified dense outer layer of PLGA may have served to prevent the easy release of BSA during both SFEE and drug release, so the EE and sustained release properties such as the low IBR could have been improved at 80 bar or 85 bar rather than at above 85 bar.

**Figure 7:**
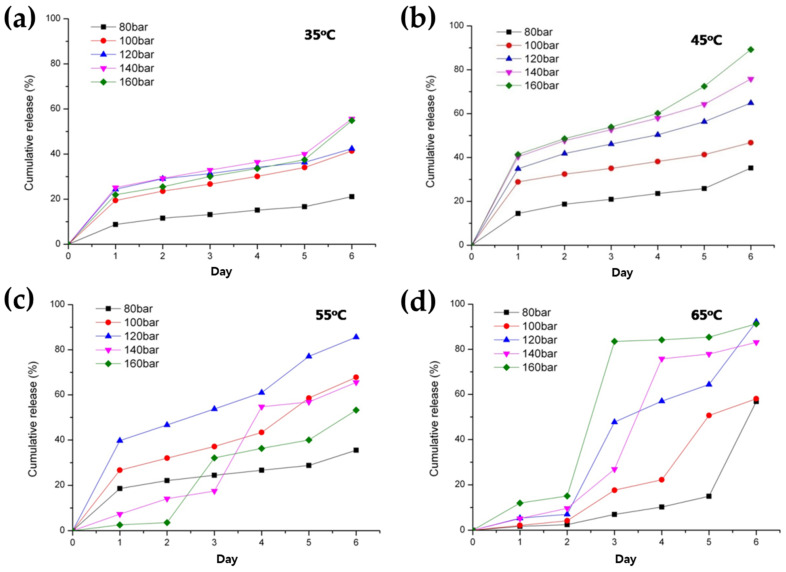
BSA release profiles from PLGA microspheres prepared via the SFEE process at various pressures and temperatures, (**a**) 35 °C, (**b**) 45 °C, (**c**) 55 °C, and (**d**) 65 °C.

**Figure 8:**
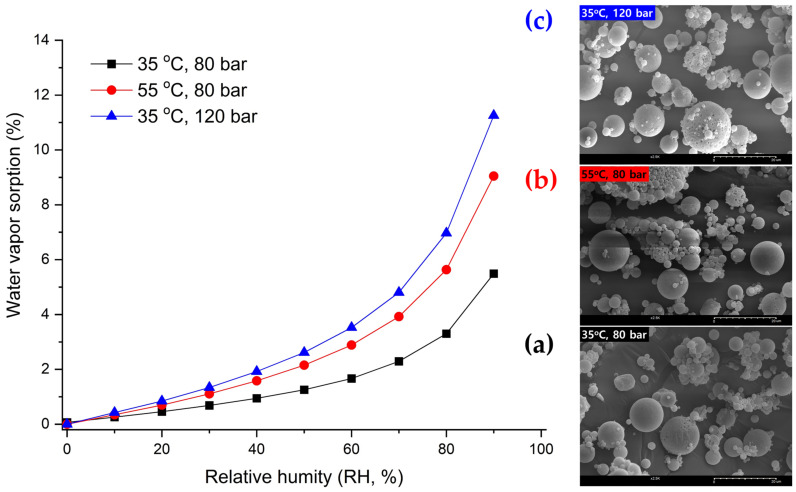
Water vapor sorption profile over increasing relative humidity (RH), and scanning electron microscopy (SEM) images of the PLGA microspheres prepared at (**a**) 35 °C and 80 bar, (**b**) 55 °C and 80 bar, and (**c**) 35 °C and 120 bar.

It was observed that the IBR value exhibited a tendency to increase with rising pressure at both 35 °C and 45 °C, contrary to the trend observed for EE (Figure 6c) [53]. For more information, BSA release profiles from PLGA microspheres for a time period of 6 days are presented in Figure 7. In addition, water vapor sorption was reduced with a pressure reduction from 120 bar to 80 bar at 35 °C (Figure 8). This result may indicate a dense surface and lower surface area (lower porosity) for the PLGA microspheres prepared via SFEE at 35 °C and 80 bar. This was confirmed through SEM image analysis, allowing the observation that the size and number of pores were relatively small for the PLGA microspheres prepared via SFEE at 35 °C and 80 bar. Typically, the pores of the PLGA microspheres can be formed by water channels, facilitating mass transfer through the oil layer from the internal water phase to the outer phase [45,54,55,56,57]. The high porosity of the PLGA microspheres is closely associated with the low EE [58], and also leads to an increase in IBR due to the larger surface area available for excessive drug release [59]. Based on this theory, the result of water vapor sorption explains why the EE and IBR of the BSA-loaded PLGA microspheres prepared using the SFEE process at 35 °C and 80 bar were improved compared with those of microspheres prepared at 35 °C and 120 bar. In addition, the high IBR of the drug from PLGA microspheres can also be attributed to proteins adhered to the outer surfaces that failed to be entrapped inside the microspheres and that escaped along the pore channel of the PLGA layer [4]. Consequently, in cases where the EE of the prepared microspheres was low, a higher proportion of drugs were distributed on the external surface of the microspheres, leading to a larger IBR [60,61,62]. 

In contrast, the IBR of microspheres prepared at pressures exceeding 120 bar at 45 °C was notably lower than that of other microspheres (Figure 6c and Figure 7). This finding was unexpected, given that the EE decreased with increasing pressure. This discrepancy may be attributed to the reduced surface area, resulting from particle coalescence and agglomeration under high-pressure conditions, as demonstrated in the optical microscopic images (Figure 6d).

**Figure 9:**
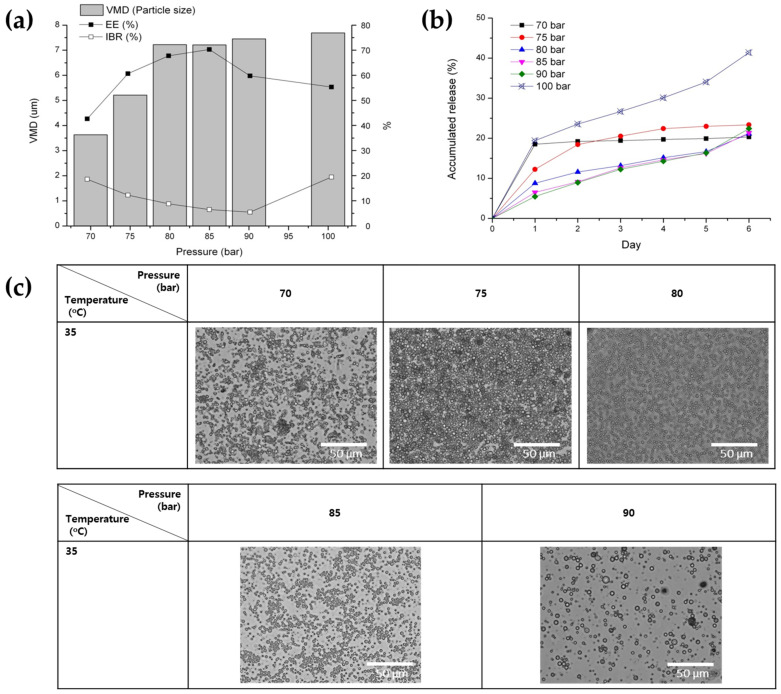
The effect of the increase in pressure from 70 to 100 bar on the properties of BSA-loaded PLGA microspheres prepared via the SFE process at 35 °C: (**a**) the particle size, EE, IBR, (**b**) drug release profile for 6 days, and (**c**) morphology observed via optical microscopy.

To investigate in more detail the impact of pressure variations on the notable alterations in the characteristics of PLGA microspheres, observed by the change in process pressure from 80 bar to 100 bar at 35 °C, additional experiments were conducted under pressure conditions ranging from 70 to 90 bar. Interestingly, a decrease in pressure below 80 bar actually resulted in a decrease in the particle size of the microspheres (Figure 9a,c). These results can be elucidated by referring to a previously documented theory indicating that at a low pressure, the volume of droplets initially increases until it reaches a maximum value, and subsequently decreases until a dense thin layer of the polymer is formed [46]. On the other hand, EE decreases and IBR increases as pressure decreases below 80 bar (Figure 9a). It is hypothesized that this outcome can be attributed to the delayed solidification of the outer layer of the microsphere, resulting from the lower DCM extraction efficiency at pressures below 80 bar at 35 °C. This phenomenon may lead to a decrease in EE through the outward diffusion of proteins across the oil layer during the solvent removal process, accompanied by an increase in IBR (Figure 9a,b).

#### 3.2.2. Effect of Temperature

It was observed that the particle size of the microspheres exhibited a notable increase as the temperature increased (Table 2 and Figure 6a). This outcome can be elucidated by considering the phenomenon wherein the rate of shrinkage of the dichloromethane (DCM) droplets diminishes at elevated temperatures. This deceleration occurs due to both the rapid diffusion of SC-CO_2_ into the DCM droplet and the outward diffusion of DCM from the emulsion to the surrounding aqueous phase, facilitated by the high temperature surpassing the boiling point of DCM. Thus, the excessive expansion of the DCM droplets occurs at high temperatures, and the fabrication of PLGA microspheres occurs too quickly before complete droplet shrinkage caused by the high DCM removal efficiency created via the combined effect of extraction and evaporation, leading to a larger particle size of the obtained PLGA microspheres. This decreased shrinking rate and excessively increased evaporation of DCM can lead to a decrease in the density and more porous internal structure of microspheres (Figure 8).

In a similar fashion to the decrease in EE observed with increasing pressure as discussed previously, EE likewise decreased with rising temperature (Figure 6b). The low EE due to the formation of the porous surface and microsphere breakage could also be associated with the high internal pressure of the hydrated microspheres at a higher SFEE process temperature than the T_g_ of PLGA. In addition, particle agglomeration at high temperatures may also be another cause of the particle size increase (Figure 6d and Figure 8). It was supposed that the increase in particle size above 35 °C at 85 bar could have increased on a more flexible surface, hence resulting in particle agglomeration in the suspension [1].

As anticipated, BSA-loaded microspheres with decreased EE exhibited an increase in IBR as the temperature increased. Similar to other processes for manufacturing PLGA microspheres, which rely on the principle of solvent evaporation and extraction, an elevation in temperature during SFEE can also induce the formation of large pores as a result of the excessive generation of vaporized gas. This estimate was validated as an actual result through water vapor sorption analysis, as shown in Figure 8.

#### 3.2.3. Effect of Stirring Rate and SC-CO_2_ Flow Rate

**Figure 10:**
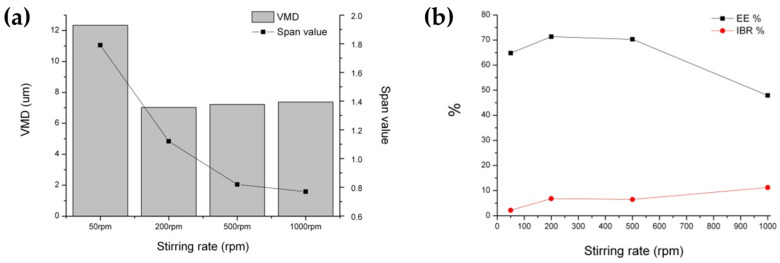
The effect of stirring rate during the SFEE process on the properties of BSA-loaded PLGA microspheres prepared via the SFE process at 35 °C and 85 bar; (**a**) particle size and distribution; (**b**) EE and IBR.

**Figure 11:**
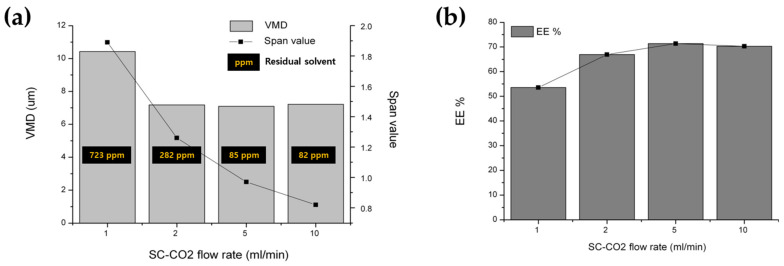
The effect of the SC-CO_2_ flow rate during the SFEE process on the properties of the BSA-loaded PLGA microspheres prepared via the SFE process at 35 °C and 85 bar; (**a**) particle size and distribution; (**b**) EE and IBR.

It was observed that the particle size and SPAN value increased, while the EE decreased, at low stirring rates or SC-CO_2_ flow rates (Figure 10 and Figure 11). The proposed rationale for this decrease in extraction efficiency was the delayed formation of the outer shell and/or the less compact outer layer of the PLGA microspheres, potentially leading to the loss and diffusion of the inner drug into the outer phase. This low mixing/extraction efficiency could have also led to the slow formation of a dense thin layer of PLGA, thereby promoting the greater coalescence of emulsion droplets. In addition, the higher extraction efficiency at an increased SC-CO_2_ flow ratio resulted in lower residual solvent content in the PLGA microspheres [26,63].

## 4. Conclusions

As double-emulsion manufacturing process parameters, the primary (W/O) and secondary emulsion (W/O/W) homogenization speed and secondary emulsification time were evaluated. In addition, the effect of the SFEE process parameters, including pressure (70–160 bar), temperature (35–65 °C), stirring rate (50–1000 rpm), and flow rate, of SC-CO_2_ (1–40 mL/min) on PLGA microsphere quality properties were also evaluated. An increase in the homogenization speed of the primary emulsion resulted in an increase in EE and a decrease in IBR. In contrast, increasing the secondary emulsification speed resulted in a decrease in EE and an increase in IBR along with a decrease in microsphere size. The insufficient secondary emulsification time resulted in excessive increases in particle size, and excessive durations resulted in decreased EE and increased IBR. Increasing the temperature and pressure of SFEE resulted in an overall increase in particle size, a decrease in EE, and an increase in IBR. It was observed that at low stirring rates or SC-CO_2_ flow rates, there was an increase in particle size and SPAN value, while the EE decreased. Overall, when the EE of the prepared microspheres was low, a higher proportion of drugs was distributed on the external surface of the microspheres, resulting in a larger IBR. A W/O/W double emulsion is a thermodynamically unstable system; thus, a shorter procedure time in this stage, that is, a rapid solidification of the double emulsion droplets, will undoubtedly favor a higher EE. Moreover, the rapid solidification of double emulsion droplets indicates a short contact time between proteins and the organic solvent, which favors the stability of the entrapped proteins. Additionally, rapid solidification can efficiently reduce the coalescence of inner aqueous droplets within oil droplets, forming a less interconnecting channel. Thus, a low initial burst and constant drug release can be expected. In this way, this study suggests that PLGA microspheres of approximately 7–8 μm in size can be manufactured by identifying the influence of process variables across the entire process from emulsion preparation to the SFEE stage. Furthermore, it was shown that drug release characteristics can be controlled by altering the process variables. In contrast, as mentioned in the introduction, several previous studies were limited to studying the effects of changes in formulation, without understanding the effects of the process parameters of SFEE. As a result, a previous paper reported that BSA containing PLGA microspheres in the range 1–4 µm, smaller than the generally accepted particle size of PLGA microspheres for sustained release, were manufactured without an evaluation of EE or drug release [28].

In conclusion, this study contributes to the scientific understanding of the influence of SFEE process variables on PLGA microspheres. Therefore, based on this study, an SFEE process with high extraction efficiency could be developed as a desirable PLGA microsphere-manufacturing technology.

## Figures and Tables

**Table 1:** Change in process parameters of double emulsion preparation and their effects on the emulsion and PLGA microsphere.

No.	Primary (W/O) Emulsion				Secondary (W/O/W) Emulsion				Temperature (°C)	d_sd_/d_pd_^c^	Microsphere			
	Homogenization Speed (rpm)	Time (min)	VMD ^a^ (µm)	SPAN ^b^ Value	Homogenization Speed (rpm)	Time (min)	VMD ^a^ (µm)	SPAN ^b^ Value			VMD ^a^ (µm)	SPAN ^b^ Value	EE ^d^ (%)	IBR ^e^ (%)
1-1	6500	5	10.34	0.97	6500	1	28.38	1.27	35	2.74	18.05	1.84	6.25	67.41
2-1	9500	5	5.80	0.92	6500	1	26.37	1.36	35	5.50	14.25	1.72	13.63	49.70
3-1	13,500	5	3.02	0.81	6500	1	25.92	1.24	35	8.58	14.80	1.63	21.45	32.59
4-1	17,500	5	1.23	0.84	6500	1	23.86	1.05	35	19.43	15.44	1.15	24.79	33.93
5-1-0.5	21,500	5	-	-	6500	0.5	60.88	1.86	35	83.36	42.39	2.32	49.62	61.30
5-1	21,500	5	0.73	0.79	6500	1	24.01	0.99	35	32.87	13.25	1.17	46.01	25.26
5-1-2	21,500	5	-	-	6500	2	22.65	0.81	35	31.02	12.65	0.94	24.79	40.86
5-1-4	21,500	5	-	-	6500	4	21.29	0.84	35	29.15	12.95	0.91	20.55	41.37
5-2	21,500	5	-	-	9500	1	16.44	1.14	35	22.51	10.03	0.99	21.94	26.08
5-3	21,500	5	-	-	13,500	1	9.09	1.02	35	12.45	5.64	0.92	15.14	39.83
5-4	21,500	5	-	-	17,500	1	6.61	0.94	35	9.05	3.65	0.98	12.47	56.05
6-1	24,000	5	0.75	0.72	6500	1	23.29	0.91	35	31.05	12.97	1.05	44.28	27.92

^a^ Volume mean diameter; ^b^ value calculated as the ratio of (D_90%_–D_10%_) to D_50%_, where DN% indicates the volume particle diameter at each cumulative volume percentage; ^c^ ratio of the droplet diameter (d_sd_/d_pd_) of the secondary emulsion (d_sd_) against that of the primary emulsion (d_pd_); ^d^ encapsulation efficiency; ^e^ initial burst release measured on the first day.

**Table 2:** The applied SFEE conditions (of pressure and temperature) and quality properties of PLGA microspheres prepared via SFEE.

Temperature (°C)	Pressure (bar)	VMD ^a^ (μm)	EE ^b^ (%)	IBR ^c^ (%)
35	70	3.63	42.65	18.52
	75	5.21	60.66	12.23
	80	7.22	67.75	8.78
	85	7.21	70.28	6.49
	90	7.45	59.75	5.47
	100	7.69	55.34	19.41
	120	7.26	52.59	24.39
	140	8.17	45.12	25.13
	160	8.70	32.64	21.98
45	80	7.73	60.66	14.47
	100	7.89	34.21	28.88
	120	8.26	28.67	34.88
	140	8.77	27.63	40.35
	160	8.98	23.70	41.41
55	80	8.35	58.89	18.58
	100	8.87	31.53	26.67
	120	9.03	29.94	39.70
	140	9.84	22.37	7.35
	160	9.79	30.78	2.55
65	80	9.77	43.82	1.73
	100	9.82	32.56	2.13
	120	9.89	17.3	5.32
	140	9.86	17.93	5.12
	160	9.98	18.66	11.98

^a^ Volume mean diameter, ^b^ encapsulation efficiency, and ^c^ initial burst release measured on the first day.

## Data Availability

The data presented in this study are available in this article.
